# Sociodemographic predictors of flourishing among older adults in rural and urban Mongolia

**DOI:** 10.1038/s41598-023-28791-x

**Published:** 2023-01-31

**Authors:** Saranchuluun Otgon, Sugarmaa Myagmarjav, Denise Burnette, Khorolsuren Lkhagvasuren, Fabio Casati

**Affiliations:** 1 The School of Public health, Mongolian National University of Medical Sciences, Ulaanbaatar, 14210 Mongolia; 2 School of Social Work, Virginia Commonwealth University, Richmond, 23284 USA; 3 Department of Information Engineering and Computer Science, University of Trento, Trento, 38122 Italy

**Keywords:** Psychology, Psychology and behaviour

## Abstract

Flourishing is an eudaimonic dimension of psychological well-being associated with positive social and health outcomes. Determining correlates of health and well-being is critical for the development of evidence-based best practices, policies, and action plans that target older adults, especially in low- and middle-income countries where research on ageing, health and well-being is still scarce. The study aimed to determine the level of flourishing among older adults in Mongolia and to explore demographic and social factors that contribute to their flourishing in urban and rural areas of Mongolia. We used proportional quota sampling to select a non-probability sample of 304 community-dwelling older adults that reflected the national distribution of older age groups and rural/urban residency. We adapted and administered a widely used standardized questionnaire on flourishing and used multiple regression to establish correlates of flourishing. Study participants reported “very high” levels of flourishing; differences in median scores 53 for urban and 50 for rural older adults were significant. Sex ($\beta =-\,2.09$, $p=0.080$), level of education($\beta =0.98$, $p=0.009$) and receive help with ADLs ($\beta =-\,2.29$, $p=0.013$) were associated with flourishing in rural areas, as were self-rated health ($\beta =1.14$, $p=0.028$), number of social activities ($\beta =0.050$, $p=0.019$),and friends network ($\beta =0.22$, $p=0.035$) in urban areas. Despite facing many challenges to well-being, older adults in Mongolia reported high levels of flourishing. Those in urban areas had higher scores than those in rural areas and predictors of flourishing differed for these groups.

## Introduction

The improvement of population well-being is a growing societal goal^1^ that is motivated by demographic trends such as population aging and robust evidence that links psychosocial factors to health and well-being^2^. The development of evidence-based practices, programs and policies that target older adults thus requires the determination of factors that contribute to their physical health and psychological well-being^3^. However, research on these topics remains scarce in resource-constrained settings^4^. In Mongolia, research initiatives that target older adults are limited to health and social welfare issues, with only a few studies addressing aspects of more general well-being^5–7^. As a result, there is little knowledge about the nature and scope of diversity in this sector of the population or about factors that affect their well-being. This gap can hinder or preclude the ability to develop and implement evidence-based policies and services that will effectively address the specific needs of this growing sector of Mongolian society. Mongolia is the most sparsely populated nation in the world and for many centuries the dominant lifestyle was nomadic pastoralism^8^. Even today, one-third of the population lives nomadic or semi-nomadic lives, residing in “aimags” (provincial centers) or “soums,” the smallest unit of government administration, outside the capital city of Ulaanbaatar (aimag subsidiaries)^9^. Since the collapse of the Soviet Union in 1991, norms and social practices of nomadic pastoralists have changed with massive rural-to-urban migration, as young adults seek emerging economic opportunities in and around Ulaanbaatar^10^. Low incomes and limited access to health care^11^ and social services in rural areas^12^ have spurred this migration to urban and peri-urban areas. According to the most recent national statistical report of the Mongolian population, only about 30% of older adults live in the countryside^6^. As younger family members relocate to larger urban areas to access opportunities for work and health and social services^7^, many older adults are ‘left behind’ with inadequate pensions^13^, poor infrastructure and little or no access to high quality care, while needing to cope with extremely harsh weather during long seasons of the year^14^. The current study examines correlates of psychological well-being among community-dwelling older adults living in urban and rural areas of Mongolia. More specifically, we focus on flourishing as the primary measure of psychological well-being. Originating in the work of Ryff^15^ and Keyes^16^, flourishing is defined as a eudaimonic dimension of subjective well-being, which represents an individual’s overall evaluation of how well they are functioning in the world^17^. The concept derives from well-established theories of optimizing life in the process of aging, and it has been studied extensively since the early 2000s. A key finding from this body of research is that high levels of flourishing are reliably associated with positive social and health-related outcomes among older adults^18–20^.

## Methods

### Sampling and study setting

This cross-sectional study was conducted between October and December 2016. According to the National Statistical Office Census Report, 131,043 older adults (females aged 55+ and males aged 60+ as defined by the “Law on Elders”) live in Ulaanbaatar city and 163,928 in the countryside. Of these 294,971 older adults, 70% were women. Using the proportional quota sampling shown in Table 1, we recruited 304 community-dwelling older adults (210 females and 96 males) who met the age criterion and had been living for at least one year at their current address (Table 1).

**Table 1:** Proportional quota sampling strategy.

Age group	Total population	Study sample	Urban	Rural
55–59	115,125	125	68	58
60–64	65,496	71	39	32
65–69	43,089	47	25	22
70+	71,976	78	42	36
Total	295,686	322	174	148

Six trained interviewers with backgrounds in social work and public health conducted face-to-face interviews of about 30 minutes in duration in elder associations, geriatrician offices, and respondents’ homes. Additional time was allowed for participants who grew tired or had a significant hearing impairment. Respondents received a 10,000 MNT (  4 EUR) compensation for participating.

### Variables and measures

The survey instrument comprised measures of flourishing, social networks and social activities, self-rated health, and demographic characteristics.

- *Flourishing* was measured using a well-validated 8-item scale developed by Diener et al.^21^, that is based on psychological well-being theories^22,23^. Respondents are asked whether they have a purposeful and meaningful life; have supportive and rewarding relationships; are engaged and interested in activities; contribute to the happiness and well-being of others; feel competent in important activities; perceive themselves as a good person; feel optimistic; and feel that others respect them. Items are measured on a 7-point Likert scale ranging from strongly disagree = 1 to agree strongly = 7 (theoretical range 8 to 56), and higher scores indicate higher levels of psychological resources and strengths. We used standard forward and back translation protocols to translate the instrument from English to Mongolian and pretested it with 20 older adults at a rehabilitation center in Ulaanbaatar. The Cronbach’s alpha was 0.77.
- *Social network* We measured social networks with a validated Mongolian version^24^ of the LSNS-6^25^, the short version of the Lubben Social Network Scale. Each of the two subscales contains three items that assess the size, closeness, and frequency of contact with family and friends. The total score is an equally weighted sum of the six items. Questions are scored 0 to 5 (theoretical range of 0 to 30), and higher scores indicate more robust networks. Cronbach’s alpha was 0.77 for the overall scale, 0.82 for family, and 0.75 for friend subscales.
- *Social activity* was assessed by a series of questions from the social participation component of the Survey of Health, Ageing and Retirement in Europe (SHARE)^26^. The questions ask if and how often the respondent has engaged in 7 types of volunteer, social, and cognitively stimulating activities and their reasons for participation. Responses are summed to obtain the frequency of social participation (theoretical range 7-28). High scores indicate high levels of social participation.
- *Internet usage* The survey included a series of questions on technology and internet usage based on the Pew Internet Survey^27^. Due to the low level of Internet penetration in the target population, we included a single question in our analyses: “Do you use the internet, at least occasionally?”
- *Health and autonomy*. We use the single self-rated health item to measure the perceived health status^28^. Response options were poor = 1, fair = 2, good = 3; very good =4 and excellent = 5. We also asked whether participants receive help with activities of daily living (ADL)^29^. Response categories were yes = one and no = 0.
- *Sociodemographic* measures are drawn from the Survey of Health, Ageing and Retirement in Europe (SHARE)^26^. They included age, sex (female = 2; male =1 ), area of residence (urban = 1; rural = 2), marital status (married or living with a partner = 1, single or divorced = 2, and widowed = 3), family size (number of people who live in the same household), paid work (yes = 1; no = 0 ) during the last month. A retirement year (number of years) is included. Education was classified as: illiterate or primary =1, secondary = 2, high school = 3, vocational school = 4, and university degree = 5.

### Statistical analyses

We used SPSS version 19 for Windows to analyze the data. We first derived descriptive statistics for all sociodemographic, health, and social functioning variables. Numeric variables were expressed as median, interquartile and categorical variables were expressed as percentages and frequencies. To examine differences between rural and urban subsamples we used the Kruskal-Wallis test for numerical variables and the Chi-Squared test for categorical variables. Finally, we used OLS multiple regression to assess correlates of flourishing. We performed diagnostics to ensure that assumptions were met. The multi-tolerance criteria indicated no multicollinearity among the independent variables.

### Ethical approval

The Mongolian National University of Medical Sciences Ethical Review Board approved the study on May 27, 2016 (protocol 15:32016-15).

### Consent and accordance

We confirm that all methods were performed in accordance with the relevant guidelines and regulations. All participants signed informed consent documents.

## Results

### Descriptive statistics

Table 2 presents descriptive statistics of study participants by urban/rural status. The median age of the participants was 62 (*IQR* = 58–69). General demographic, health, and most social wellbeing measures were comparable for respondents in the two geographic areas. Urban older adults reported higher education, larger family size, and greater internet usage and those in rural areas reported more social activities. The median score for flourishing in the overall sample was 52, meaning that the vast majority of participants reported “very high” psychological resources and strengths^21^. The median flourishing score among urban older adults (Mdn = 53) was significantly higher than that of their rural counterparts (Mdn = 50, $p = 0.001$) (Table 2).

### Predictors of flourishing

We used multiple linear regression models to estimate the effects of age, sex, education level, marital status, retirement years, self-rated health, ADL assistance, family size, frequency of social activities, LSNS-6 (friends and family subscales), and internet use on flourishing. We analysed rural and urban subsamples separately and with the combined population sample for reference (Table 3).

The regression model for the rural subsample accounted for 43.4% of the variance in the flourishing score ($F(14,122) = 6.620, p < 0.000, R2 = 0.433$). Significant predictors were sex (female), higher education, being employed, better self-rated health, not receiving help for ADLs, having a larger family size, and having more contact with friends on the friends sub-scale of the LSNS-6. In the urban subsample, predictors accounted for only 19.3% of the variance ($F(14,142) = 2.226, p = 0.009, R2 =0.183$). Better self-rated health, more social activities and more contact with friends on the LSNS sub-scale contributed significantly to flourishing scores. Self-rated health and the LSNS-6 friends subscale were the only significant contributors to flourishing in both rural and urban areas. Finally, receiving help with activities of daily living significantly contributed to flourishing of older adults. Taken together, our results suggest that older adults in Mongolia who live in rural settings experience a greater number of factors that negatively affect their likelihood of flourishing in later life than those who live in urban areas.

**Table 2:** Descriptive statistics.

	Overall	Urban	Rural	P-Value
Sample size	304	162	142	
Age (median[IQR])	62 [58.00, 69.00]	62.00 [58.00, 69.00]	63.00 [58.00, 70.00]	0.631$^a$
Sex = Female (%)	210 (69.1)	112 (69.1)	98 (69)	0.982$^b$
Marital status (%)				0.102$^b$
Married/partner	146 (48.03)	87 (53.7)	59 (43.5)	
Divorced/single	17 (5.6)	6 (3.8)	10 (7)	
Widowed	141 (46.4)	68 (42)	73 (51.4)	
Education (%)				<**0.000**$^b$
Illiterate/primary	48 (15.8)	7 (4.3)	41 (29.1)	
Secondary	53 (17.5)	17 (10.5)	36 (25.5)	
High school	79 (26.1)	46 (28.4)	33 (23.4)	
Vocational	53 (17.5)	36 (22.2)	17 (12.1)	
University	70 (23.1)	56 (34.6)	14 (9.9)	
Self-rated health (median[IQR])	3.00 [3.00, 4.00]	3.00 [2.00, 3.00]	3.00 [2.00, 3.00]	0.296$^a$
Receive help for ADL = no (%)	212 (71.1)	116 (74.3)	96 (67.6)	0.199$^b$
Paid work = Not paid (%)	256 (84.4)	137 (84.6)	119 (83.8)	>0.855$^b$
Retirement years (median [IQR])	9.00 [4.00, 21.00]	9.5 [4.00, 19.5]	9.00 [4.00, 22.00]	0.883$^a$
N. Social activities (median [IQR])	3.00 [2.00, 4.00]	2.00 [1.00, 4.00]	4.00 [3.00, 4.00]	<**0.000**$^a$
Family size (median [IQR])	2.00 [2.00, 4.00]	3.00 [2.00, 5.00]	2.00 [1.00, 4.00]	**0.009**$^a$
LSNS (median [IQR])	18.00 [14.00, 23.00]	18.00 [14.00, 23.00]	19.00 [15.00, 23.00]	0.087$^a$
LSNS family (median [IQR])	9.00 [7.00, 12.00]	9.00 [7.00, 12.00]	10.00 [7.00, 12.00]	0.154$^a$
LSNS friends(median [IQR])	9.00 [6.00, 12.00]	9.00 [5.00, 12.00]	10.00 [7.00, 12.00]	0.144$^a$
Use Internet = Yes (%)	63 (21)	49 30.2)	14 (10.1)	<**0.000**$^b$
Flourishing (median [IQR])	52.00 [47.00, 55.00]	53.00 [48.00, 55.00]	50.00 [47.00, 54.00]	**0.002**$^a$

Difference between samples computed using (a) Kruskal-Wallis for continuous variables non normally distributed, and (b) Pearson Chi-squared test for categorical variables.

**Table 3:** Socio-demographic determinants of flourishing among Mongolian older adults by area of residence.

	Overall			Urban			Rural		
	Estimates	CI	P-value	Estimates	CI	P-value	Estimates	CI	P Value
(Intercept)	45.13	37.23–53.04	<**0.000**	48.19	37.50–58.88	<**0.000**	34.44	22.20–46.70	<**0.000**
Age	− 0.38	− 0.16 to 0.81	0.081	− 0.60	− 0.22 to 0.10	0.446	0.016	− 0.177 to − 0.21	0.871
Sex (Female)	− 1.310	− 2.73 to 0.10	0.069	− 0.988	− 2.82 to 0.84	0.288	− 2.09	− 4.43 to 0.25	**0.080**
Education level	0.56	0.90–1.03	0.020	− 0.96	− 0.76 to 0.57	0.776	0.98	0.25–1.71	**0.009**
Marital status (Married)									
Divorced Single	− 1.38	− 3.88 to 1.11	0.276	− 3.06	− 6.79 to 0.68	0.108	0.81	− 2.87 to 4.49	0.664
Widowed	0.34	− 0.86 to 1.55	0.575	0.61	− 0.95 to 2.18	0.439	0.298	− 1.67 to 2.27	0.765
Paid work (Not paid work)	1.69	0.128–3.25	**0.034**	0.90	− 1.36 to 3.16	0.432	1.97	− 0.29 to 4.22	**0.087**
Retirement years	0.06	− 0.02 to 0.15	0.161	0.03	− 0.09 to 0.15	0.593	0.08	− 0.05 to 0.20	0.239
Self-rated health	1.01	0.23–1.78	**0.011**	1.14	0.13–2.16	**0.028**	1.37	0.04–2.70	**0.043**
Receive help for ADL (No)	− 0.95	− 2.22 to 0.32	0.141	0.39	− 1.47 to 2.25	0.681	− 2.29	− 4.09—0.50	**0.013**
No. social activities	0.35	0.04–0.67	**0.025**	0.50	0.08–0.92	**0.019**	0.13	− 0.37 to 0.62	0.614
Family size	0.26	− 0.02 to 0.53	0.065	0.03	− 0.34 to 0.39	0.884	0.51	0.08 to 0.94	**0.019**
LSNS family	0.07	− 0.10 to 0.23	0.440	− 0.02	− 0.27 to 0.23	0.888	0.11	− 0.13 to 0.35	0.380
LSNS friends	0.28	0.13–0.44	**0.001**	0.22	0.02–0.43	**0.035**	0.43	0.17–0.69	**0.001**
Use Internet (Yes)	0.13	− 1.63 to 1.38	0.866	0.30	− 1.52 to 2.12	0.743	− 0.59	− 3.45 to 2.28	0.686
Living area (Urban)	− 1.86	− 3.17 to − 0.55	** 0.006**						
Observations	290			154			136		
R2/adjusted R2	0.288/0.249			0.183/0.114			0.434/0.368		

*Bold text indicates statistically significant predictors.

## Discussion

This study advances current knowledge on circumstances that enable older adults to thrive in later life. Our findings may also be relevant to findings on other global measures of well-being, e.g., quality of life and subjective well-being, in identifying factors that contribute to optimizing aging. Variables associated with flourishing represent key focal areas for practitioners and policymakers. We will highlight several areas of significance in this regard. *First*, it is important to note that study participants reported “very high” levels of flourishing, or psychological resources and strengths. Older Mongolian adults thus appear to regard their lives positively in areas such as relationships, self-esteem, purpose, and optimism. To put these results into context, the few previous studies with older adults worldwide have reported varying levels of flourishing. Surveys with European older adults report mean scores ranging from 39.2 in Finland^30^, 46.7 in Croatia^31^, and in Mongolia’s neighboring country, the Russian Federation^32^ a mean score was 40.9. The few studies among older adults in Southeast Asia and Oceania, report an average flourishing score of 50.16 for Malaysian older adults^33^, and of 45.19 among New Zealanders^34^. Given these results, the median flourishing score observed in this study ($Mdn=52$) is the highest but closer to those reported in the survey of older adults in another Asian country. Diener and colleagues describe flourishing as related to cultural features^35^, with individualistic cultures emphasizing personal autonomy and motives, while in collectivist cultures the group is more important than the individual. They note that people in more individualistic cultures tend to make internal attributions for events and are more likely to follow their interests and desires to pursue self-fulfillment^36^.

Indeed, individualists report higher levels of happiness than collectivists and they score higher in flourishing and mental health^37^. Diener and colleagues^36^ also note that higher levels of flourishing hold consistently for certain East Asian nations, where people are more likely to sacrifice positive emotions to achieve other goals that they deem essential. Considering that Mongolian culture is more individualistic than collectivist^38^, there are some theoretical grounds for our results. However, the lack of representative studies on the flourishing of the older adult population prevents us from drawing definitive conclusions.

Another potential explanation for high levels of flourishing in our sample is the positive life rule of Buddhist doctrine. According to the Buddhist attitude of mind, saying bad things or good things is related to destiny. Buddhism is one of the most critical influences on Mongolian culture^39^, with approximately half of the population following Tibetan Buddhism and Shamanism/Tengrism^10^. These attitudes or predispositions may challenge the operational definition of flourishing, making comparisons more tenuous. A second main contribution of this study is the identification of correlates of flourishing in the study population. Employed older adults had higher levels of flourishing in the total sample, but employment was slightly more important for those living in rural areas. This finding is consistent with same-aged adults in other Asian countries, including Malaysia^33^ and Eastern Thailand^40^. There are two reasons why employed older adults’ flourishing level might be lower than the unemployed in the Mongolian context. *First*, as noted, rural poverty has led to major internal migration to the Ulaanbaatar area, which in turn has deepened urban poverty. There are no social safety nets in rural or urban areas and herders and older adults who live in *soum* (district) and aimag (province) centers have limited access to education, health care, infrastructure, information, jobs, and few human development and economic opportunities^41^. Another reason for the effects of employment is the low income of older adults. In 2016, the National Statistical Office reported an average retirement pension of 117.4 dollars^6^, which is insufficient to cover household and livelihood expenses in Mongolia. Self-rated health is a strong predictor of subjective well-being for older adults^1^, and it was significantly associated with flourishing in rural and urban areas. Similar findings have been reported in Malaysia^33^, Thailand^40^, and Russia^42^. Satisfaction with health falls rapidly with age in the populations of post-Soviet countries, including Mongolia, in part because of poor quality health care^43^. Differences in the health infrastructure and social services^7^ and the type of labor associated with rural life can significantly limit individuals’ health and autonomy in these locales. Closeness and frequency of contacts with friends was also a significant factor in both rural and urban populations. Older adults with stronger friend networks reported higher levels of flourishing than those with a smaller friend network. Studies on psychological well-being consistently report that social ties and social contacts are vital to human thriving^44,45^. Robust networks of friends and kin are highly valued throughout Mongolia^10^, where kinship and local community culture stem from historic social patterns of nomadic herding cultures ($hot-ail$). *Finally*, social activity was significantly associated with flourishing for older adults in the urban setting. As noted, social activities such as paid work, volunteer work, care of family members, and informal help to friends among older adults enhance positive feelings of self-efficacy, self-esteem, and self-worth and contribute to a sense of mastery and personal control, autonomy, and self-determination^46^. For example, productive activities such as providing help and volunteering are associated with positive well-being among older adults in European countries^47^. Hank et al.^48^ also point out that positive societal images of aging, a longstanding feature of Mongolian society and religious traditions, can influence older adults’ participation in productive activities. Our findings advance knowledge of rural/urban differences in older adults’ flourishing in several ways. Those living in urban areas reported higher levels of flourishing ($Mdn=53$) than those in rural areas ($Mdn=50$). This finding is at odds with previous studies in similar settings mentioned above. Place of residence was not related to flourishing in Malaysia where, unlike Mongolia, living conditions are equivalent in rural and urban areas^33^. It is thus necessary to consider migration patterns^6^, availability of infrastructure, health, and social services^7,49^, adequacy of retirement benefits^13^ and even weather conditions^14^. Sociodemographic factors also influence flourishing differently in rural and urban areas. Sex and education made a difference in rural areas, which is in line with previous studies^33,40,50,51^. It may be that men and women differ in their understanding, evaluation, and reflection of what matters in life^52–55^. Although the education level was lower in rural than urban areas, overall levels were high. This finding is due to social and political initiatives under the People’s Republic of Mongolia, which invested in education to improve the understudy generation’s access to primary and higher education^56^. Education level was associated with flourishing only in rural areas, which differs from the previously mentioned study with older Malaysians^33^. The authors of that study surmised that the effects of education might interact with other social and economic variables. On the other hand, Diener et al.^36^ suggest that education may not be related to psychological well-being. Interestingly, family size, but not the more complex social network construct that assesses closeness and frequency of contacts, was a significant predictor of flourishing in rural settings. At the same time, as in previous studies, personal and family-related factors were unrelated to flourishing in our urban subsample. Family relationships were essential to older adults’ well-being in Thailand^40^, networks with intimate people were necessary to older adults’ survival in Siberia^42^, the absence of children’s support created problems for older Koreans^57^, and a number of children were a strong predictor of well-being for older adults in Japan^50^. In Mongolia, family relationships are central, and the aforementioned rural-to-urban migration of younger generations highlights the adverse effects on older adults in rural areas^6^. Receiving assistance with activities of daily living was also a strong predictor of flourishing for rural older adults. This finding is interesting since we included only older adults living independently in the community. Diene et al.^36^ suggest that levels of support depend on social norms and are typically high in collectivist cultures. Pinquart et al.^52^also point out that the type of help received affects older adults’ well-being. For example, social and emotional support was determined to be essential for older adults to cope with life crises in Siberia^42^. In our study, this finding may be attributable to the participants’ traditional social group. Most older adults in rural areas are former herders who have adapted to the traditional informal collective action of *hotail*, whereby a small group of family and friends help one another care for livestock^58,59^. This social pattern may also account for assertive hospitality behavior among rural people in Mongolia^60^.

### Limitation

 This study has several limitations. First, participants were recruited from health centers and elders’ associations in which participants are registered. This might create a selection bias. There also may be possible seasonal effects as we collected the data between November and December. As this is the coldest part of the year in Mongolia, results may have thus been affected by weather conditions^60^. Finally, as data were cross-sectional, we could not assess individual-level or population-level change in levels of flourishing.

## Conclusion

Flourishing scores were higher among older adults in urban areas than in rural areas; however, Mongolian older adults overall reported relatively high levels of flourishing. Determinants differed for older adults in urban and rural areas. Self-reported health, number of social activities, and friend networks were associated with flourishing in urban areas. At the same time, sex, level of education, and receipt of ADL assistance contributed to flourishing in rural areas. Our findings provide evidence to inform the development and implementation of targeted policies, programs, and practices based on shared and unique social and demographic factors in rural and urban areas.

## Data Availability

The datasets used and analyzed during the current study are available from the corresponding author upon reasonable request.
